# Exploring Changes in the Microbiota of *Aedes albopictus*: Comparison Among Breeding Site Water, Larvae, and Adults

**DOI:** 10.3389/fmicb.2021.624170

**Published:** 2021-01-28

**Authors:** Francesca Scolari, Anna Sandionigi, Martina Carlassara, Antonia Bruno, Maurizio Casiraghi, Mariangela Bonizzoni

**Affiliations:** ^1^Department of Biology and Biotechnology, University of Pavia, Pavia, Italy; ^2^Department of Biotechnology and Biosciences, University of Milano–Bicocca, Milan, Italy

**Keywords:** breeding site, water, larvae, 16S rRNA gene, *Wolbachia*

## Abstract

The mosquito body hosts highly diverse microbes, which influence different physiological traits of both larvae and adults. The composition of adult mosquito microbiota is tightly linked to that of larvae, which are aquatic and feed on organic detritus, algae and prokaryotic microorganisms present in their breeding sites. Unraveling the ecological features of larval habitats that shape the structure of bacterial communities and their interactions with the mosquito host is still a poorly investigated topic in the Asian tiger mosquito *Aedes albopictus*, a highly invasive species that is vector of numerous arboviruses, including Dengue, Chikungunya, and Zika viruses. In this study, we investigated the composition of the bacterial community present in the water from a natural larval breeding site in which we separately reared wild-collected larvae and hatched eggs of the Foshan reference laboratory strain. Using sequence analysis of bacterial 16S rRNA gene amplicons, we comparatively analyzed the microbiota of the larvae and that of adult mosquitoes, deriving information about the relative impact of the breeding site water on shaping mosquito microbiota. We observed a higher bacterial diversity in breeding site water than in larvae or adults, irrespective of the origin of the sample. Moreover, larvae displayed a significantly different and most diversified microbial community than newly emerged adults, which appeared to be dominated by Proteobacteria. The microbiota of breeding site water significantly increased its diversity over time, suggesting the presence of a dynamic interaction among bacterial communities, breeding sites and mosquito hosts. The analysis of *Wolbachia* prevalence in adults from Foshan and five additional strains with different geographic origins confirmed the described pattern of dual *w*AlbA and *w*AlbB strain infection. However, differences in *Wolbachia* prevalence were detected, with one strain from La Reunion Island showing up to 18% uninfected individuals. These findings contribute in further understanding the dynamic interactions between the ecology of larval habitats and the structure of host microbiota, as well as providing additional information relative to the patterns of *Wolbachia* infection.

## Introduction

The microbiota of *Aedes* mosquitoes is known to play a significant role in host physiology, including egg production, blood digestion (Gaio Ade et al., 2011; Coon et al., 2016a), immunity regulation (Xi et al., 2008), host–pathogen interaction and vector competence (Ramirez et al., 2012; Charan et al., 2013; Goncalves et al., 2014; Dickson et al., 2017; Souza-Neto et al., 2019).

The microbiota of both laboratory and wild *Aedes* mosquitoes has been investigated and shown to be dominated by Proteobacteria (Wang et al., 2018). However, despite the overall composition of the microbiota was similar between laboratory-reared and wild mosquitoes (David et al., 2016), the diversity of midgut bacterial communities was found to be higher in field-caught mosquitoes (Osei-Poku et al., 2012). In *Aedes aegypti*, different bacterial communities were detected between domestic and sylvatic habitats (Dickson et al., 2017). Moreover, when *Ae. aegypti* populations from the field were reared in the laboratory, they were shown to display a similar midgut microbiota (Dickson et al., 2018). These findings indicate the importance of the environment in shaping mosquito microbiota.

Mosquitoes are holometabolous insects with larval and adult stages occupying different ecological niches, thus exploiting different resources. Larvae develop in aquatic habitats, while adults are terrestrial. Larval stages acquire their symbionts primarily through feeding in their breeding site water, with their microbiota representing a subset of the bacteria found in the water (Coon et al., 2014, 2016b; Dada et al., 2014; Dickson et al., 2017; Wang et al., 2018). Adults can introduce bacteria through feeding on nectar (Merritt et al., 1992), imbibing water from their breeding sites at emergence, as occurring in *Anopheles gambiae*, or *trans*-stadially from larval gut bacterial during metamorphosis (Lindh et al., 2008). Thus, larval and adult stages are not independent from each other and biotic and abiotic features of the larval environment can influence adult microbiota.

The Asian tiger mosquito *Aedes albopictus* (Skuse, 1894) is a highly invasive species of growing public health concern due to its ability to transmit at least 22 arboviruses, including Chikungunya, Dengue, and Zika viruses (ISSG Invasive Species Specialist Group, 2020; Lwande et al., 2020). The worldwide expansion of this species was favored both by anthropogenic factors such as increased mobility and trades, and biological characteristics of this species, such as its ability to undergo photoperiodic diapause and use natural and artificial breeding sites. A number of studies have described the microbiota of *Ae. albopictus* in recent years, including the prevalence of *Wolbachia* (Zouache et al., 2009; Chouaia et al., 2010; Minard et al., 2013; Valiente Moro et al., 2013; Minard et al., 2014, 2015; Yadav et al., 2015, 2016; Coon et al., 2016b; Park et al., 2016; Ahmad et al., 2017; Muturi et al., 2017; Mancini et al., 2018; Rosso et al., 2018; Thongsripong et al., 2018; Wang et al., 2018; Guegan et al., 2018; Hu et al., 2020; Mancini et al., 2020).

*Wolbachia* is a genus of Gram-negative bacteria infecting about 40% of arthropod species (Zug and Hammerstein, 2012) and almost 30% of mosquito species (Ricci et al., 2002). *Wolbachia* is transovarially transmitted and is able to exert a number of reproductive manipulations, such as cytoplasmic incompatibility, that can be exploited for the control of mosquito populations (Floate et al., 2006; Bourtzis et al., 2014). Moreover, the generation of novel *Wolbachia* transinfections in *Ae. aegypti* showed to have an impact on host susceptibility for several pathogens (see for example Moreira et al., 2009; Bian et al., 2010; Walker et al., 2011; van den Hurk et al., 2012; Frentiu et al., 2014; Ye et al., 2015; Ant et al., 2018). *Aedes albopictus* transinfected with the *w*Mel *Wolbachia* strain is unable to transmit Dengue (Blagrove et al., 2012) or Chikungunya (Blagrove et al., 2013) viruses in laboratory assays. *Aedes albopictus* is naturally superinfected with two *Wolbachia* strains, *w*AlbA and *w*AlbB (Dutton and Sinkins, 2004). Thus, to develop effective *Wolbachia*-based strategies for controlling *Ae. albopictus*, it is essential to assess the stability and population invasion potential for *Wolbachia* newly generated infections. Because this is most likely linked to *Wolbachia* inter-strain interactions (Ant and Sinkins, 2018), it important to determine *w*AlbA and *w*AlbB prevalence across *Ae. albopictus* populations and strains.

In *Ae. albopictus*, most studies focused on the characterization of adult endosymbionts, and little is known about larval microbiota and how it can shape adult biological traits. Additionally, the complexity of the interactions between the mosquito host and the bacteria in breeding sites and the relationships between *Wolbachia* infection and other components of the microbiota are still poorly understood.

In this exploratory study, we aimed at expanding the current understanding of the dynamic interactions between the ecology of larval habitats and the structure of host microbiota, as well as the patterns of *Wolbachia* infection. To do so, we addressed three main questions: (i) to what extent is the breeding site microbiota affecting endosymbiont community assemblage in larval and adult *Ae. albopictus*? (ii) Is the bacterial community of the breeding site water changing over time during mosquito development? (iii) What is the frequency of *Wolbachia* in adult samples reared as larvae in the wild-collected water?

To answer these questions, we compared bacterial community composition among (i) water from natural larval breeding sites, (ii) mosquito larvae, and (iii) adult individuals. In water from a natural breeding site, we reared both wild-collected larvae and larvae of the Foshan reference strain to derive information about the relative impact of the environment and the genetic background of mosquitoes on shaping mosquito microbiota. Finally, we analyzed *Wolbachia* presence in Foshan and five additional laboratory strains with different geographic origins.

## Materials and Methods

### Water Sampling

Water from *Ae. albopictus* larval breeding sites was collected in a private garden in Crema, Italy (45°21′51.2′′N 9°40′57.7′′E), which was an accessible site characterized by high population density, at the end of August 2018, when climate conditions are optimal for mosquito development. Domestic collection of water, eggs, and larvae originated from two plastic buckets of a maximum volume of 500 ml that were placed in the same garden next to ornamental plants. Environmental water collected in these buckets derived from dew and rain and was monitored for its level and clarity every day for 4 days. No sedimentary layer was observed. Water and eggs/larvae were then collected in sterile 50-ml Falcon tubes and transferred to the insectary of the University of Pavia, Italy. The collected water was divided into three aliquots and used as follows: (i) an aliquot of 200 ml was brought to the ZooPlant laboratory in Milano, Italy, to characterize its microbial composition (samples hereafter called W Start); (ii) 300 ml were aliquoted in two different pans (150 ml/pan) and daily monitored for natural larval hatching in the Pavia insectary (samples named CR); (iii) 600 ml were used to hatch eggs from the Foshan strain (Chen et al., 2015; Palatini et al., 2020), after having verified the absence of wild eggs using a stereomicroscope. The Foshan strain is an established laboratory colony derived from wild mosquitoes from South-East China and reared in the Pavia insectary since 2013 (Palatini et al., 2017). A total of 400 Foshan eggs were hatched, in batches of 100, each in 150 ml of breeding site water (samples named FO). To avoid overcrowding, the amount of water was determined based on the average hatching rate of the Foshan strain in our insectary conditions. No food was added in any pan to allow larvae to grow based on the nutrients present in the breeding site water.

For both the CR and FO samples, the developing individuals were collected separately as fourth instar larvae (in pools of eight individuals) and adults (individually collected the day of their emergence). In the case of adult collections, pupae developed in the larval rearing pans were individually transferred to cups containing 10 ml of the same wild water until adult eclosion. When mosquito development was completed and all adults had eclosed, the remaining water from each rearing pan was transferred into sterile 50 ml Falcon tubes for the analysis of the microbiota (samples hereafter called W End).

All mosquito life stages were maintained in the Pavia insectary at 28°C and 80% RH, with a photoperiod of 12:12 hrs light:dark.

### Chemical Analyses of Water Samples

For each water sample, total nitrogen (N_tot), nitrites (NO_2_), nitrates (NO_3_), ammoniacal nitrogen (NH_4_-N), and phosphate (P_tot) were measured with a spectrophotometer (Spectroquant Pharo 300, Merck). Moreover, pH and conductivity were also recorded. Analyses were performed using the following kits, according to the manufacturer’s instructions: Ammonium Test Photometric Method NH_4_-N, Nitrate Test Photometric Method NO_3_-N, Nitrite Test Photometric Method NO_2_-N, Total Nitrogen Test Photometric Method, Phosphate Test Photometric Method PO_4_-P, Merck Spectroquant^®^.

### Sample Pre-processing and DNA Extraction

Genomic DNA was extracted from (i) water samples at the two time-points described above; (ii) pooled larval (L) samples; (iii) individual adult (A) mosquitoes collected immediately after eclosion.

Water samples were processed according to a modified version of a previously described protocol (Bruno et al., 2017a). First water samples were filtered by a serial vertical (orthogonal) filtration using nitrocellulose membrane filters with a pore size of 8 μm, followed by a filtration with a membrane with pores of 3 μm. Vacuum was generated by a vacuum pump (ME 2 NT VacuubrandTM) connected to a filtering apparatus. The filtrate was collected and concentrated using tangential flow filtration (TFF) to recover as much biological material as possible. The TFF system involved a peristaltic pump (Masterflex L/S Economy Drive), Tygon^®^ tubing, sterile reservoirs and filtration modules. The used tangential flow filter was a VivaFlow^®^ 200 cassette (Sartorius) made of polyethersulfone (PES) with a nominal pore rating of 10000 MWCO and a surface area of 200 cm^2^. The system was scaled up with an additional unit connected in parallel to increase the filtration surface area and the flow speed. The TFF system was run at a transmembrane pressure of 1.5 bar. The initial water samples were concentrated to a final retentate volume of 100 mL, which was further reduced to 1 mL through the Vivaspin^®^ 20 ultrafiltration unit (Sartorius) made of PES with a nominal pore rating of 10000 MWCO. The final volume was allowed to be adsorbed onto a membrane filter before DNA extraction.

All tubing, tubing connections, and containers were sterilized with sodium hypochlorite or autoclaved prior to each experiment and among samples. Every step was conducted in the laminar flow cabinet in a pre-amplification dedicated laboratory.

Environmental DNA was extracted separately from each of the filters obtained by water filtration using DNeasy^®^ PowerSoil^®^ Kit (Qiagen) with the QIAcube (Qiagen) automated system, following the manufacturer protocol. DNA was eluted in 75 μL of warmed (40°C) elution buffer, to increase DNA concentration. DNA extraction negative controls were included.

A total of 25 *Ae. albopictus* samples were processed, namely 11 larval pools (four CR and seven FO samples, respectively) and 14 individual adults (five CR, of which four males and one female; nine FO, of which six males and three females, respectively). The finding that sequencing pools of six mosquitoes allowed to capture a level of bacterial diversity comparable to that of single mosquitoes (Bennett et al., 2019), together with our aim to achieve the maximum information level, prompted us to process larvae and adults differently. Larval samples were treated as pools of eight individuals, similarly to other studies (e.g., Wang et al., 2018) to minimize the biological variability of the microbiota among individuals and, at the same time, avoid overcycling in amplicon PCR. Individual adults were also used to obtain data related to *Wolbachia* infection at the single-mosquito level. Prior to DNA extraction from both larval pools and adults, each individual was surface-washed twice with 1X PBS, after washing in ethanol 70% (following Seabourn et al., 2020). DNA extraction was performed using the Wizard Genomic DNA Purification Kit (Promega) following the manufacturer’s instructions. DNA extraction negative controls were included.

All the procedures were carried out in a laminar flow cabinet, in order to avoid contamination with exogenous DNA and inter-samples contamination, and in separate rooms for the pre- and post- amplification steps, with dedicated personal protective equipment.

### 16S Metagenomic Sequencing of Mosquito and Water Samples

Illumina MiSeq 16S libraries were generated following the standard protocol “16S Metagenomic Sequencing Library Preparation, Part # 15044223 Rev B.” Amplicon PCR was performed using PCR primers 341F (5′-CCTACGGGNGGCWGCAG-3′) and 805R (5′-GACTACHVGGGTATCTAATCC-3′) with Illumina library adaptors, for 25 cycles of amplification. DNA extraction negative controls (Bianco_1EXT, Bianco_2EXT) and amplicon PCR negative controls were included in library preparation. Libraries quantification through TapeStation 4100 (Agilent) showed no amplicon signal for amplicon PCR negative controls, which were excluded from the sequencing. In the case of *Ae. albopictus* samples, amplicon sequencing was run by Macrogen, Inc. using an Illumina MiSeq platform and the Herculase II Fusion DNA Polymerase Nextera XT Index Kit V2. Water samples were sequenced by the Center for Translational Genomics and Bioinformatics – San Raffaele Scientific Institute (Milan, Italy) with Illumina MiSeq 2 × 300 paired-end chemistry (MiSeq Reagent Kit v3).

### Illumina Data Processing and Analyses of the Microbiota Composition

The raw paired-end FASTQ reads were imported into the Quantitative Insights Into Microbial Ecology 2 program (QIIME2, ver. 2017.9.01) (Caporaso et al., 2010) and demultiplexed using native plugin. The Divisive Amplicon Denoising Algorithm 2 (DADA2) (Callahan et al., 2016) was used to quality filter, trim, denoise, and mergepair the data and remove chimeric sequences.

The resulting Amplicon Sequence Variants (ASVs) with less than a 50x coverage were discarded from further analyses. The classification of the obtained ASVs was run using the feature-classifier plugin implemented in QIIME2 against the SILVA SSU non-redundant database (138 release), adopting a consensus confidence threshold of 0.8. The analysis on the bacterial diversity as well as the corresponding figures were done using the *phyloseq* R package (McMurdie and Holmes, 2013).

Any reads assigned to *Wolbachia* were filtered using the filtered taxa plugin of QIIME2.

Microbiota diversity was described in terms of within (alpha) and between (beta) sample diversities. The Shannon index and Observed Features alpha diversity metrics were calculated to estimate the variation of bacterial diversity in the water, larvae and adult samples. Values were compared using the pairwise Kruskal–Wallis test.

To explore the bacterial diversity of our samples, we used a relative abundance bar plot to show phyla and family distribution between our three sample types (water, larvae, and adults). The distribution of the 100 most abundant ASVs was studied with a heatmap plot. Both visualizations were obtained with the *phyloseq* R package.

Rarefaction is used to subsample and calculate distances among samples. Beta diversity was estimated with quantitative distance metrics using the diversity QIIME2 plugin based on the rarefied dataset with a sampling depth of 10,000 sequences. Samples with lower depth were automatically discarded from beta diversity analysis.

We estimated the unweighted UniFrac and Bray–Curtis dissimilarity indexes by sampling 10,000 reads per sample (Lozupone et al., 2007). Statistical significance among groups, including sampling site and developmental stage, was determined by a permutation-based ANOVA (PerMANOVA) test using ADONIS (Anderson, 2005) and a 999 permutation-based UniFrac distance metrics. PerMANOVA Pairwise contrast was performed by the beta-group-significance command of *diversity* plugin. The structure of microbial communities was explored by Non-Multidimensional Scaling (NMDS), an ordination approach (Kruskal, 1964). Sequences representative of each community were aligned with MAFFT and used for phylogenetic reconstruction in FastTree (Price et al., 2010).

The list of bacterial genera, as derived from ASVs, in each sample was compared using Venn diagrams following two criteria. We considered ASVs showing more than 50 reads and occurring in at least two tested samples as ‘general’ ASVs. Then, among these ASVs, we looked for those occurring in at least 70% of all tested samples; we considered these ASVs as the “conserved” mosquito microbiota. This analysis allowed us to explore the flux of bacterial symbionts acquired from the water environment by the mosquito larvae and maintained until the adult stage. Venn diagrams were created using an online tool^1^.

### qPCR Amplification of 16S rRNA Gene in Water Samples

Quantitative Real Time PCR (qPCR) assays were performed targeting the 16S rRNA gene to verify the sensitivity of our approach in detecting the microbiota of water samples and to compare relative bacterial abundances between W Start and W End, according to a protocol previously described (Bruno et al., 2017a). Water samples, samples deriving from TFF filtrate, DNA extraction negative controls and amplification negative controls were tested in triplicates.

Cycling conditions adopted were as follows: an initial denaturation at 95°C for 10 min, 40 cycles of denaturation at 95°C for 15 s and annealing-elongation at 55°C for 1 min. A final dissociation stage was performed. Amplification reaction consisted of 5.0 μl SsoFast EvaGreen Supermix with Low ROX (Bio-Rad S.r.l., Segrate, Milan, Italy), 0.1 μl each 10 μmol l^–1^ primer solution, 2 μl DNA sample, and 2.8 μl of Milli-Q water. Assays were performed on an AB 7500 thermocycler (Applied Biosystem) and results analyzed as previously described (Bruno et al., 2017a).

### Estimates of *Wolbachia* Prevalence by PCR

To obtain a qualitative validation of *Wolbachia* infection status in the 25 adult samples used for metagenomic analysis, PCR reactions for *Wolbachia* detection were performed in 15 μl reaction volumes consisting of 7.5 μl DreamTaq Green Master Mix (Thermo Fisher Scientific, Eugene, OR, United States), 4 μl of autoclaved Milli-Q water, 1 μl primers (10 μM), and 1.5 μl of DNA (∼20 ng). The PCR cycling conditions were as follows: 3 min at 95°C for the initial denaturation step, followed by 35 cycles of 1 min at 95°C, 1 m at 55°C, 1 m at 72°C and 10 min at 72°C for the final extension. The published *wsp* primers were used (Zhou et al., 1998): 328F (5′-CCAGCAGATACTATTGCG-3′) and 691R (5′-AAAAATTAAACGCTACTCCA-3′) for the detection of *w*AlbA, and 183F (5′-AAGGAACCGAAGTTCATG-3′) and 691R for *w*AlbB. PCRs reactions were run on the same samples also using the primer set Aealbo18S_F1 (5′-TGCCATGGATGCTTTCATTA-3′) and Aealbo18S_R1 (5′-GTACAAAGGGCAGGGACGTA-3′) to test for DNA quality. All PCR products were visualized on 1% agarose gels and sequenced.

Moreover, the presence of *Wolbachia* was assessed by PCRs in Foshan (generation 37) and five additional long-established laboratory strains deriving from Canton (China, G20), Recife (Brasil, G25), Tapachula (Mexico, G31), Tampon (La Reunion, G26), and Crema (Italy, G33). These strains were selected for several reasons. First, to compare *Wolbachia* prevalence estimated through metagenomics in CR and FO samples (reared in wild-collected water) with estimates of *Wolbachia* presence in the Foshan and Crema laboratory strains. The Crema strain was established in 2016 from mosquito eggs collected in the same breeding site used in this study. Second, to determine whether insectary rearing for several generations have led to a 100% *Wolbachia* frequency in adult individuals.

## Results

The microbiota of breeding site water (W), larval (L), and adult (A) *Ae. albopictus* samples was examined by sequencing of the bacterial 16S rRNA gene. Sequences of a total of 37 libraries (*i.e.*, 10 water samples; four CR and seven FO larval samples; five CR and nine FO adults, respectively; two DNA extraction negative controls) resulted in 8,191,150 million sequence reads, ranging from 14,277 to 264,318 (with an average of 126,018). No reads were reported in negative controls. Total number of reads per sample before and after *Wolbachia*-associated reads removal is reported in Supplementary Table S1. Identified ASVs per sample ranged between 19 and 3410 (Supplementary Table S2).

**FIGURE 1:**
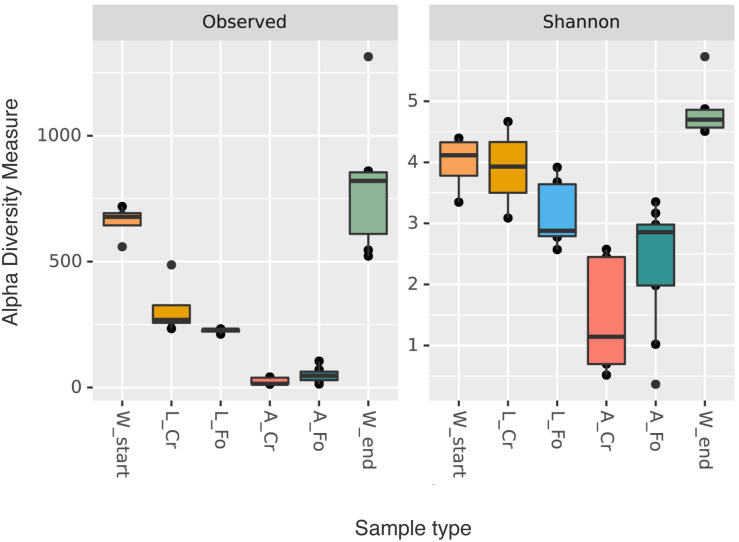
Alpha diversity indices (richness and Shannon). Box plots of alpha diversity values distribution for both indices (richness and Shannon diversity) of the water (W Start, orange; W End, light green), larval (CR, golden; FO, blue), and adult (CR, coral; FO, dark green) samples are shown. The line inside each box represents the median value. Outliers are shown as dots.

W End samples showed higher variability in the number of ASVs than W Start samples; larval samples showed a much higher variability than adults, irrespective of their origin (Figure 1). These differences are statistically supported, as shown by the results of the Kruskal–Wallis test (Table 1). Quantity of bacterial DNA in water samples was also estimated by qPCR (Supplementary Figure S1A and Supplementary Table S3) confirming a significantly higher number of bacterial DNA copies in W End in comparison to W Start (ANOVA, Tukey *post hoc* test: *p* < 0.05).

**TABLE 1:** Pairwise comparison (Kruskal–Wallis test) of diversity indices between water, larval, and adult samples.

		Water		Crema		Foshan
		W Start	W End	L_CR	A_CR	L_FO
Water	W End	**6.54 (0.010)**				
Crema	L_CR	0.083 (0.773)	3.682 (0.05)			
	A_CR	**6.00 (0.014)**	**7.50 (0.006)**	**6.00 (0.014)**		
Foshan	L_FO	**4.321 (0.038)**	**9.00 (0.003)**	2.893 (0.089)	**7.180 (0.007)**	
	A_FO	**7.384 (0.007)**	**9.60 (0.02)**	**5.654 (0.017)**	1.371 (0.242)	**1.929 (0.165)**

**H* and *p*-values (in brackets) are shown. Significant comparisons (*P* < 0.05) are indicated in bold. The number of samples within each group were: W Start (*n* = 4). W End (*n* = 6), L_CR (*n* = 4), L_FO (*n* = 7), A_CR (*n* = 5), A_FO (*n* = 9).*

Water chemistry at mosquito breeding sites has been shown to have a key impact on mosquito survival and abundance (Chen et al., 2009; Rajesh et al., 2013; Onchuru et al., 2016). Additionally, specific physical and chemical parameters (*i.e.*, oxygen and conductivity) were found to be associated with microbiota composition in *Ae. aegypti* (Hery et al., 2021). Thus, in an attempt to provide a qualitative analysis that could support the metagenomic results, a set of physical and chemical parameters were measured for both W Start and W End samples. Both pH and conductivity significantly increased in W End samples (*t*-test, *P* < 0.05) (Supplementary Figure S1B and Supplementary Table S4). Moreover, nitrates, ammoniacal nitrogen and phosphate values were higher (at least > 2.5 folds) in W End than in W Start, providing further support to the presence of differences between the two considered samples, despite measurements were often close to detection limits (Supplementary Table S4).

### Microbiota Composition and Distribution

A total of 28 bacterial phyla and 262 families were identified across all samples. Taxonomic analysis showed that most of the sequences are associated with the phylum Proteobacteria (59.7%), followed by Bacteroidota (16.4%), Firmicutes (3.7%), and Spirochaetota (3.6%).

**FIGURE 2:**
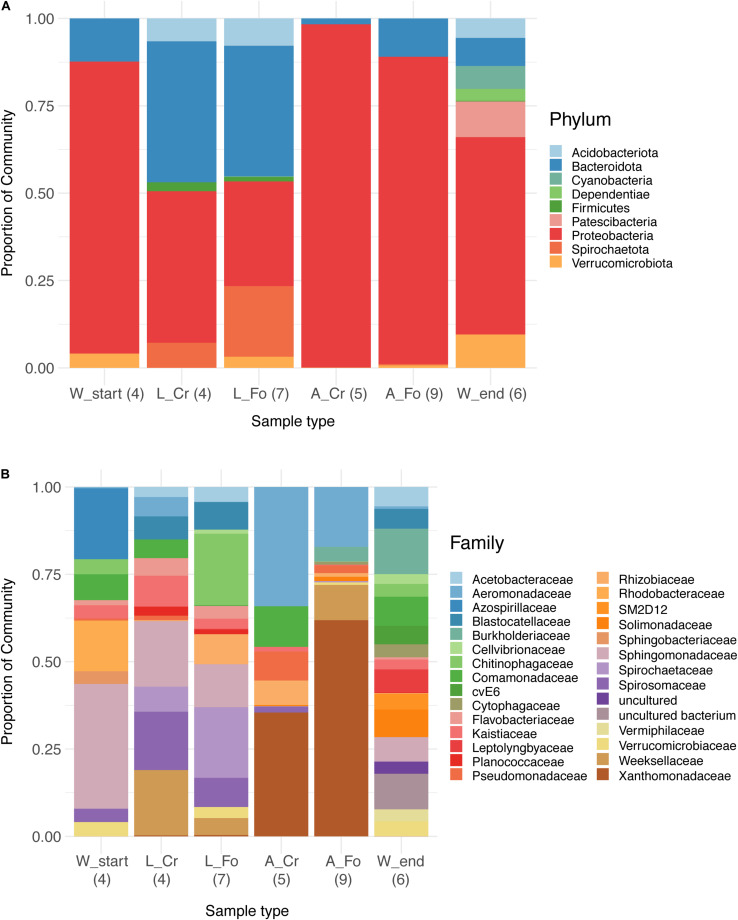
Relative abundance bar plot of bacteria at phylum **(A)** and family **(B)** taxonomy level in water, larval, and adult samples from wild-collected and laboratory mosquitoes. Each bar represents the proportion of sequencing reads (relative abundance expressed as percentage) assigned to a given bacterial taxon. Only the 50 most abundant Amplicon Sequence Variants were considered and assigned to corresponding taxa. The legend lists the most abundant taxonomic categories (9 in **A** and 29 in **B**). The number of samples analyzed for each category is reported in parentheses.

In W Start samples, the main bacterial phyla were Proteobacteria (>95%), followed by Bacteroidota (0.5%) and Verrucomicrobiota (0.3%). In addition to these phyla, in W End samples we also identified Patescibacteria, Dependentiae, Cyanobacteria, and Acidobacteria (Figure 2A). The trend of increasing diversity from W Start to W End samples is also evident at the family taxonomic level (Figure 2B). Proteobacteria and Bacteroidota dominated the microbiota of larvae; the bacterial community of adults displayed a much lower level of diversity, with Proteobacteria representing almost the totality of the microbiota of adult mosquitoes (95% in A_CR and 71% in A_FO) (Supplementary Table S5).

**FIGURE 3:**
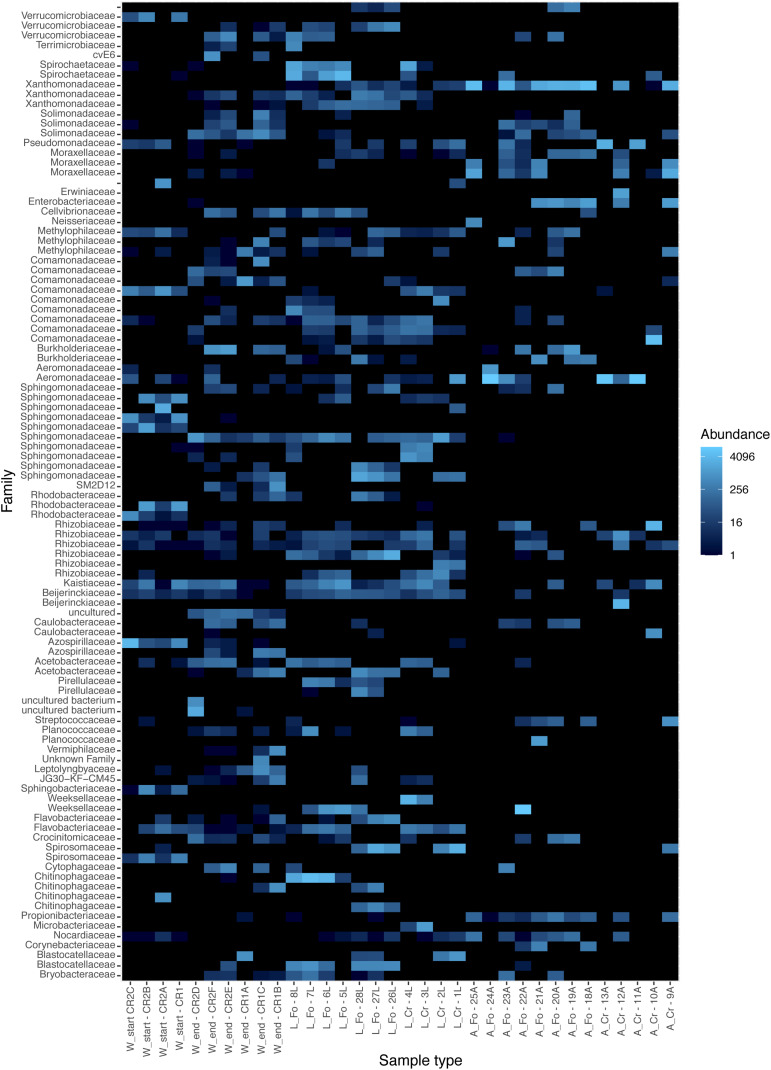
Heatmap showing the relative abundance of the components of the microbiota in the breeding site water, larvae, and adult samples from laboratory and wild-collected mosquitoes. The distribution of the 100 most abundant Amplicon Sequence Variants was explored in each analyzed sample with a heatmap plot based on rarefied tables. Each sample is shown on the *X*-axis; W refers to water samples, L and A to larvae and adult samples, respectively. Start and End refers to water collected at the moment of mosquito collection and after all adults had emerged, respectively. FO and CR refer to mosquitoes of the Foshan laboratory strain or collected in the wild, respectively. Heatmap colors (from dark to light blue) indicate increasing abundance of each microbiota component. The heatmap was generated with the *phyloseq* R package.

The trend of decreasing ASVs variability from water to adult samples is also depicted in a heatmap generated using the 100 most abundant ASVs assigned at the taxonomic level of Family (unweighted UniFrac distance) (Figure 3). The heatmap shows that the 100 most abundant ASVs are differently distributed in the water, larvae, and adult samples. Differences are evident also between W Start and W End, as well as between CR and FO samples, independently of the developmental state. At the family taxonomic level, W Start includes Sphingobacteriaceae, Spirosomaceae, Chitinophagaceae and, to a lesser extent, Nocardiaceae. W End samples include Cellvibrionaceae, Burkholderiaceae, Caulobacteraceae, Planococcaceae, Cytophagaceae, Blastocatellaceae, and several unculturable unidentified bacteria. Larval samples are characterized by Weeksellaceae, Spirosomaceae and Chitinophagaceae (phylum Bacteroidetes), Spirochaetaceae (phylum Spirochaetes), Sphingomonadaceae and Rhodobacteraceae (phylum Proteobacteria) (Figures 2B, 3).

Wild and laboratory larval samples differentiated by the presence of Verrucomicrobiaceae, Chitinophagaceae, Pirellulaceae, Cellvibrionaceae, and Terrimicrobiaceae exclusively in laboratory samples. The only bacterial family uniquely present in wild larval samples was Microbacteriaceae, with the *Herbiconiux*, *Leifsonia*, and *Leucobacter* genera.

In addition to bacteria already known to be part of the microbiota of *Aedes* mosquitoes, such as *Sphingomonas* and *Chryseobacterium* (see Scolari et al., 2019 for a review), in Foshan larval samples we also identified bacteria of the *Paenibacillus* genus, which were previously associated only with *Ae. aegypti* (Scolari et al., 2019). In Crema samples, genera reported for both *Ae. aegypti* and *Ae. albopictus*, such as *Bacillus, Pseudomonas*, and *Escherichia–Shigella*, were also found.

In the case of adult samples, the families Verrucomicrobiaceae, Acetobacteracea, Planococcaceae, Weeksellaceae, Crocinitomicaceae, Cytophagaceaea, Corynebacteriaceae, Blastocatellaceae, and Bryobacteriaceae were typical of the Foshan laboratory samples, with Weeksellaceae and Planococcaceae being particularly abundant.

Based on these results, beta diversity metrics were computed to further explore differences among samples. For more reliable results we used a rarefaction process to resample the data. Obtained rarefaction curves are shown in Supplementary Figure S2.

**FIGURE 4:**
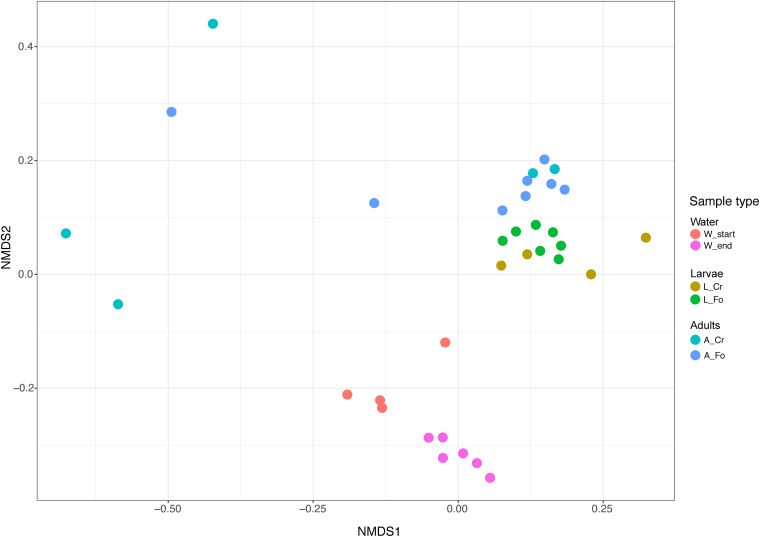
Non-metric multidimensional scaling (NMDS) of water, larval and adult samples. Colors in the bidimensional NMDS plot are used according to the different sample origin as shown in the legend. Start and End refers to water collected at the moment of mosquito collection and after all adults had emerged, respectively. FO and CR refer to mosquitoes of the Foshan laboratory strain or collected in the wild, respectively.

Samples separated into clusters in a non-metric multidimensional scaling (NMDS) ordination plot (Figure 4). Using pairwise weighted UniFrac distance matrix, the obtained stress value was <0.1. W Start and W End samples clustered together, although into two identifiable groups. All larval samples clustered together, suggesting a limited contribution of the genetic background in shaping larval microbiota. On the opposite, adult samples showed greater intra and inter sample variability, with a portion of them not being clearly separated from the larvae. PerMANOVA pairwise comparison based on the unweighted UniFrac distance metrics revealed statistically significant differences among the groups (Table 2).

**TABLE 2:** PerMANOVA pairwise comparison based on unweighted UniFrac distance metrics between water, larval, and adult samples.

		Water		Crema		Foshan
		W Start	W End	L_CR	A_CR	L_FO
Water	W End	**2.884 (0.006)**				
Crema	L_CR	**2.718 (0.023)**	**3.497 (0.007)**			
	A_CR	**3.230 (0.013)**	**4.540 (0.003)**	**2.421 (0.048)**		
Foshan	L_FO	**3.700 (0.008)**	**4.870 (0.001)**	1.330 (0.094)	**3.279 (0.002)**	
	A_FO	**3.760 (0.003)**	**5.621 (0.001)**	**2.483 (0.002)**	1.537 (0.163)	**2.9351 (0.001)**

*Pseudo-*F* and *p*-values (in brackets) are shown. Significant comparisons (*P* < 0.05) are indicated in bold. The number of samples within each group were: W Start (*n* = 4), W End (*n* = 6), L_CR (*n* = 4), L_FO (*n* = 7), A_CR (*n* = 5), A_FO (*n* = 9).*

### Sample-Unique Bacteria

**FIGURE 5:**
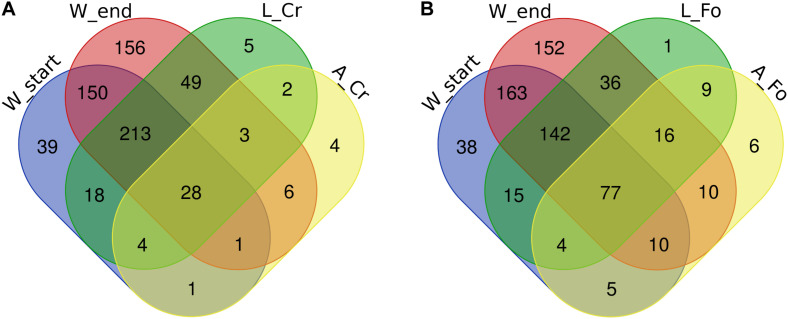
Number of unique bacterial genera in breeding site water (W), larval (L), and adult (A) samples from wild (CR) or laboratory (FO) samples. Venn diagrams show the number of shared genera in **(A)** Crema (CR) and **(B)** Foshan (FO) samples with respect to what observed in breeding site water collected at the beginning (start) or after all adults had emerged (end). The Venn diagrams were created using an online tool (http://bioinformatics.psb.ugent.be/webtools/Venn/).

The intersections among the list of bacterial genera found in analyzed samples were calculated to identify the components of the microbiota that are unique of each sample (Figure 5). As expected according to the previously described data, the number of genera shared between larvae and water samples were higher than those shared between larvae and adults, both in the case of Crema and Foshan samples (Figure 5). Wild larvae unique genera included *Dietzia*, *Blautia*, and *Leifsonia*; in wild adults, unique genera were *Tepidimonas*, *Cloacibacterium*, *Haemophilus*, and *Neisseria*. An unidentified uncultured bacterium from the family Rhodobacteraceae was found to be unique of Foshan larvae; *Dietzia*, *Meiothermus*, *Porphyromonas*, *Blautia*, *Micrococcus*, and *Fructobacillus* were the unique genera in Foshan adults.

When focusing on comparison between W Start and W End, and in both larvae and adults between FO and CR samples, the most striking result was the number of ASVs uniquely detected in adults of Foshan, and including genera such as *Prosthecobacter*, *Solimonas*, *Ancylobacter*, *Anaerococcus*, *Schlesneria*, and *Micrococcus* (Supplementary Figure S3).

### Conserved Microbiota

**FIGURE 6:**
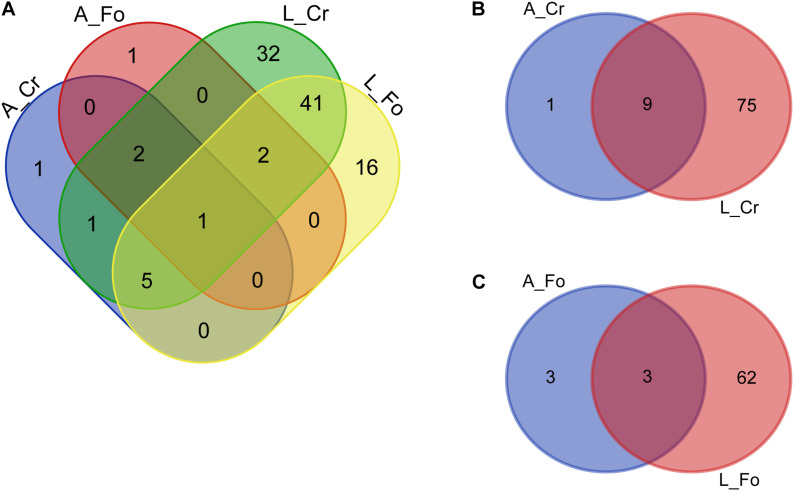
Number of unique bacterial genera from the conserved microbiota of larval (L) and adult (A) samples from wild (CR) or laboratory (FO) mosquitoes. Venn diagrams show the number of conserved genera in **(A)** adults and larvae from Crema (CR) and Foshan (FO), **(B)** within CR mosquito samples, and **(C)** within FO mosquito samples. The Venn diagrams were created using an online tool (http://bioinformatics.psb.ugent.be/webtools/Venn/).

We define “conserved” microbiota the bacterial genera detected in at least 70% of all our mosquito samples. Mosquito conserved microbiota includes 102 genera (Figure 6 and Supplementary Tables S6, S7). A total of 81 genera were also detected in water samples (*i.e.*, W Start), emphasizing the role of the breeding site in shaping mosquito microbiota. Among the bacteria absent in water samples (both W Start and W end), *Vulcaniibacterium* was the only present in both larvae and adults, of both CR and FO samples. *Escherichia–Shigella* was instead found only in CR adult samples. Several unclassified bacteria were found to be conserved in larval samples and/or adults, absent from the W Start samples but detected in the W End samples (Supplementary Table S7).

### *Wolbachia* Abundance Impacts Microbial Richness in *Ae. albopictus* Adults

**FIGURE 7:**
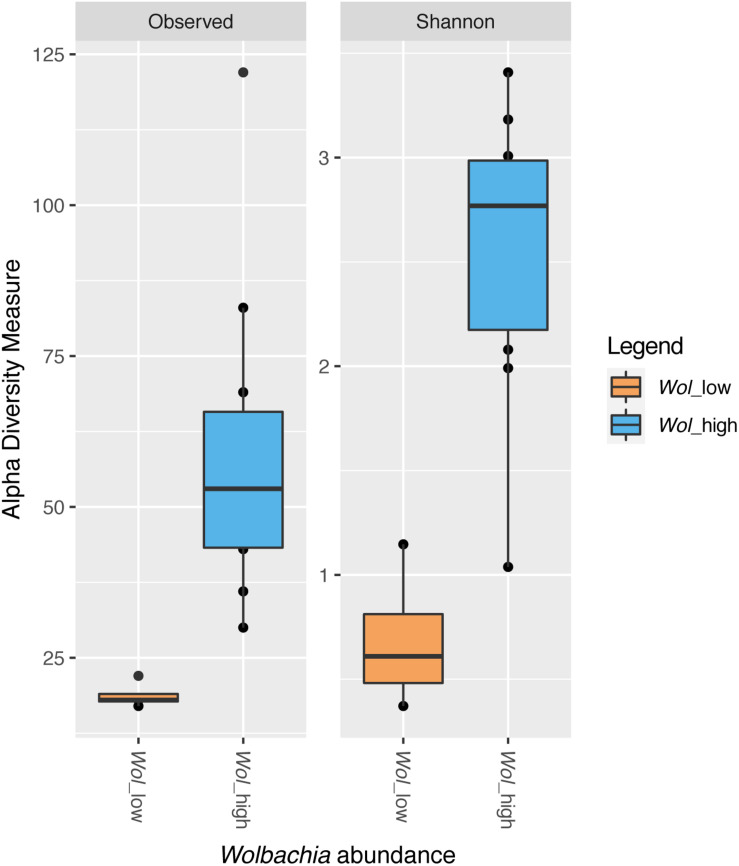
Alpha diversity indices (richness and Shannon) in samples with low (*Wol*_low, in orange) or high (*Wol*_high, in blue) *Wolbachia* abundance. *Wol*_low grouped five samples (11A_wild_CR, 13A_wild_CR, 22A_FO, 10A_Wild_CR, and 24A_FO), which had individually a Shannon diversity index < 1.5, as shown in Supplementary Figure S4, and a frequency of *Wolbachia* reads up to 41.2%. *Wol*_high grouped all the other samples, which had a frequency of *Wolbachia* reads > 86.8% and an individual Shannon diversity index > 2 (Supplementary Figure S4). The line inside each box represents the median value. Outliers are shown as dots. *Wol*_high samples displayed a significantly higher number of ASVs than *Wol*_low samples (*t*-test, *P* < 0.05).

Shannon diversity index was calculated for each sample resulting into two groups. One group included five samples (11A_wild_CR, 13A_wild_CR, 22A_FO, 10A_Wild_CR, and 24A_FO) with Shannon diversity index < 1.5; all the other samples had a Shannon diversity index higher or equal to 2 (Supplementary Figure S4). Interestingly, samples from the first group had lower frequency of *Wolbachia* reads (ranging from 0% in 11A_wild_CR and 13A_wild_CR to 41.2% in 22A_FO) than samples of the second group, thus we called these two groups “*Wol*_low” and “*Wol*_high,” respectively, and we compared their alpha diversity after excluding *Wolbachia* reads. *Wol*_high samples displayed a significantly higher number of ASVs than *Wol*_low samples (*t*-test, *P* < 0.05) (Figure 7).

### *Wolbachia* Presence in Different Laboratory Strains

The absence of *Wolbachia* in four adult samples prompted us to further investigate *Wolbachia* prevalence in six long-established laboratory strains. The prevalence of *w*AlbA and *w*AlbB strains was also determined. In all mosquito strains, both *w*AlbA and *w*AlbB were detected. However, while *Wolbachia* was present in all tested individuals in Foshan, Canton, and Recife strains, some individuals were shown to not carry *Wolbachia* in Crema, Tapachula, and Tampon (Table 3). The percentage of *Wolbachia*-negative mosquitoes ranged between 3% in Crema to 18% in Tampon. Absence was more prevalent for *w*AlbA than *w*AlbB. Patterns of *Wolbachia* infections were also evaluated in relation to mosquito sex (Table 3). Dual infection was more common in females than in males in Foshan, Canton, and Recife strains, while in Crema and Tampon, males had the highest values of infection. Furthermore, *w*AlbA-only infection was never detected in males, while *w*AlbB-only infection appeared to be mostly limited to males.

**TABLE 3:** *Wolbachia* prevalence in six *Ae. albopictus* laboratory strains.

		*Wolbachia* prevalence (%)				
		
Strain (generation)	N. samples	*w*AlbA	*w*AlbB	*w*AlbA + *w*AlbB	Total *Wolbachia*+	Total *Wolbachia* –
Foshan (G37)	40	0	22	78	100	0
	(22M, 18F)	(0M, 0F)	(22M, 0F)	(33M, 45F)		
Canton (G20)	40	0	10	90	100	0
	(20M, 20F)	(0M, 0F)	(10M, 0F)	(40M, 50F)		
Recife (G25)	40	5	10	85	100	0
	(20M, 20F)	(0M, 5F)	(10M, 0F)	(40M, 45F)		
Crema (G33)	40	0	0	97	97	3
	(20M, 20F)	(0M, 0F)	(0M, 0F)	(50M, 47F)	(50M, 47F)	(0M, 3F)
Tampon (G26)	40	5	5	72	82	18
	(20M, 20F)	(0M, 5F)	(2.5M, 2.5F)	(42M, 30F)	(45M, 37F)	(5M, 13F)
Tapachula (G31)	40	5	22	65	92	8

*Patterns of infection in relation to the sex were evaluated in positive samples for Foshan, Canton, Recife, Crema, and Tampon. M, males; F, females.*

## Discussion

### *Aedes albopictus* Larvae Acquire Microbiota From Breeding Site Water

The analysis of the diversity of bacterial communities showed that *Ae. albopictus* larvae contain a subset of the ASVs present in breeding site water, supporting the idea that larvae are colonized by a fraction of the bacteria ingested through feeding (Minard et al., 2013; Coon et al., 2016b; Strand, 2018). Higher ASVs richness in water than in mosquito samples has been already described in *Aedes* spp. (Dada et al., 2014; Dickson et al., 2017; Wang et al., 2018; Alfano et al., 2019). Among the bacterial genera present in the breeding site water and acquired by both CR and FO larvae, it is worth mentioning *Chryseobacterium*, which is known to localize in the larval gut (see Scolari et al., 2019 for a review). We also observed differences between CR and FO larvae in bacterial genera shared with W Start samples, although all larval samples grouped together in the NMDS plot, suggesting a limited contribution of the genetic background in shaping larval microbiota. For example, Foshan larval samples shared with W Start bacteria such as *Roseococcus, Paenibacillus*, and *Ferruginibacter*, which were not detected in Crema larvae. The microbiota of Crema larvae showed *Bacillus*, which was previously detected in *Ae. aegypti* (Koneman et al., 1992) and *Ae. albopictus* field-collected larvae (Coon et al., 2016b), and *Ensifer*, *Sphingobium*, and *Aeromicrobium*.

### Microbial Community of Breeding Site Water Changes Over Time

Despite the intrinsic impossibility to provide a comparison with respect to breeding site prior to mosquito oviposition (*i.e.*, a ‘natural’ control, characterized by total absence of mosquito eggs and larvae), the comparison between W Start and W End water samples suggests the presence of a dynamic interaction between larvae and their breeding site. Such a difference was also detected by qPCR amplification of the 16S rRNA gene, which confirmed a significantly higher number of bacterial DNA copies in W End in comparison to W Start. Although unculturable candidate phyla are likely present in our water samples, the procedure of relying on 16S rRNA quantification to infer bacterial load in breeding site water and mosquito samples is routinely used (e.g., Charan et al., 2013; Wang et al., 2018).

Future studies comprising water samples collected in breeding sites before mosquito oviposition will provide important data to clarify the impact of mosquito larvae on shaping the water bacterial communities. Such studies will require the possibility to robustly predict the likelihood of a water collection to become a mosquito breeding site. Moreover, additional studies comprising water controls collected at breeding sites and maintained without larval samples over time to cover the mosquito developmental window will provide further ground to investigate the actual modifications induced by larval development to the water microbial communities.

Mosquitoes are able to modify their aquatic habitat through larval feeding and excretion of bacteria from the digestive tract, an effect that can be magnified in small volume containers (Coon et al., 2016b), as adopted in our experiments. Mosquitoes are impacting their breeding sites through excretion of relatively high levels of ammonium (NH_4_^+^) from the larval anal papillae, as shown in *Ae. aegypti* (Donini and O’Donnell, 2005; Weihrauch et al., 2012). The chemical analysis of the W End samples is in line with this finding, since we detected fourfold higher levels of ammonia than in the W Start samples.

Competition among larvae may impact breeding site microenvironment and consequently affect the composition of the water microbiota over time. Competition driven by excreted chemicals has indeed been shown to occur in *Ae. aegypti* (Bédhomme et al., 2005). Larval feeding and excretion in the breeding water may contribute to developing the optimal conditions for the growing of bacteria that, in the initial water samples, could not find the ideal environment for proliferation and may thus have gone undetected. The composition of detritus-associated microfauna is indeed known to be altered over time as a consequence of larval grazing in *Aedes triseriatus* (Walker et al., 2010). Moreover, similarly to what occurs for *Wolbachia*, which is transmitted vertically from mother to offspring via the egg cytoplasm (Werren, 1997), other bacteria can be vertically transmitted (Moran et al., 2008). This can be the case of certain bacterial genera identified in our study that are not present in the water at the breeding sites, thus not being acquired through feeding. For example, *Escherichia–Shigella* was found to colonize ovaries of *An. gambiae* (Mancini et al., 2018). In *Ae. albopictus*, the reported wide dominance of *Wolbachia* in the ovaries (94% according to Mancini et al., 2018), could have masked the identification of other bacteria, which may have an importance in the biology of the species. Further studies aimed at localizing the larval- and/or adult-specific bacteria in mosquito tissues will be essential to clarify this aspect.

When considering the composition of the W End samples, it is worth mentioning the presence of Patescibacteria, which were previously shown to be associated with different developmental stages of *Ae. albopictus* (Qing et al., 2020) and adult *Culex nigripalpus* females (Duguma et al., 2019). Patescibacteria are prevalent in water environments (Tian et al., 2020), especially in groundwater (Bruno et al., 2017b; Schwab et al., 2017), and due to a reduced genome and consequent limited biosynthetic capabilities, the presence of members of this phylum may depend on nutrient uptake from other members (autotroph) of the microbial community (Brown et al., 2015; Herrmann et al., 2019). Members of this phylum have been associated with oligotrophic environments (Herrmann et al., 2019). This feature could explain the presence of members of this phylum in our water, since we allowed the larvae to grow solely based on the nutrients present in the water collected at the breeding site, which may have become depleted as a consequence of feeding. Another phylum that characterized W End samples is Dependentiae that, similarly to Patescibacteria, are widespread across different environments, including wastewater (McLean et al., 2013; Yeoh et al., 2015), and have limited metabolic capacities, suggesting their dependence on other aquatic autotrophic and heterotrophic microorganisms (Deeg et al., 2019). The abundance of Cyanobacteria is in general not surprising, since these bacteria are known to be present in mosquito breeding sites and have been previously isolated from mosquito guts (Thiery et al., 1991; Vazquez-Martinez et al., 2002). Members of this phylum are particularly abundant in environments with high phosphate concentrations (Roldán and Ramírez, 2008), which is the case of the water collected at the end of our experiments. Moreover, the growth of cyanobacteria has been shown to be favored by increase concentrations of nitrates and ammonia (Kim et al., 2017), which in our experiments could be related to mosquito development. Acidobacteria have been previously found in mosquito breeding sites (Onchuru et al., 2016), as well as adult individuals [e.g., *Culex nigripalpus* (Duguma et al., 2019), *An. coluzzii* (Mancini et al., 2018), *An. gambiae* (Mwadondo et al., 2017), *An. stephensi* (Kalappa et al., 2018), but also *Ae. albopictus* (Wang et al., 2018)].

We also identified bacteria, such as Nocardiaceae, that are shared between W Start samples and all tested mosquito samples (*i.e.*, both CR and FO larvae and adults) but they are absent in the W End sample. This pattern may indicate that members of this family are acquired and digested. In triatomines, Nocardiaceae play the role of nutritional symbionts able to provide the host with essential nutrients (Salcedo-Porras et al., 2020). Conversely, Burkholderiaceae were present in larval and adult individuals, as well as the W End, but not in W Start. Burkholderiaceae were previously found to be associated with *Ae. albopictus* breeding sites (Shelomi, 2019) as well as adults (Seabourn et al., 2020). Similarly, larval and pupal samples from *Ae. koreicus* were found to be dominated by this bacterial family (Alfano et al., 2019).

The presence of mosquito larvae has been previously shown to have different impacts on the composition of the bacterial community of breeding site water. For example, microcosmos-based experiments involving *Cx. restuans* and *Ae. triseriatus* showed that larvae affect the composition of the bacterial community in breeding sites and reduce bacterial abundance, diversity and richness (Walker et al., 1991; Muturi et al., 2020); a similar trend was seen in natural breeding sites in tree holes for *Ae. triseriatus* (Walker et al., 1991). These results contrast with those obtained by Kaufman et al. (1999) in the same mosquito species, in a microcosms-based set-up, as they found that larval presence increase total bacterial numbers, similarly to the increased bacterial abundance detected in water columns in small container habitats reported for the pitcher plant mosquito *Wyeomyia smithii* (Heard, 1994; Cochran-Stafira and von Ende, 1998).

Larvae can contribute to the development of enriched and more anoxic conditions, which favor the growth of facultative anaerobe bacteria.

### Newly Emerged *Ae. albopictus* Adults Display a Simplified Microbiota

The data obtained in this study show a decrease in microbiota complexity from larval to adult stage. This trend, evident in both wild and laboratory samples, is most likely due to the dramatic changes the gut epithelium undergoes during metamorphosis. In particular, the formation of two meconial peritrophic matrices (MPM1 and MPM2) was shown to contribute to *Ae. aegypti* adult midgut sterilization by sequestering microorganisms ingested during the larval stage, which are excreted after emergence (Moll et al., 2001; Moncayo et al., 2005). This process is not fully understood in *Ae. albopictus.*

After such loss of components, adults shape a new microbiota and diet plays a key role. Mosquito hosts, such as plants and animals, are a source of bacteria, as well as viruses and other microorganisms. Indeed, it is known that the midgut of the two sexes harbors different microbial communities: the microbiota of females is typical of blood feeding insects, and it is mostly colonized by Gammaproteobacteria; males, instead, display a midgut mainly colonized by Firmicutes (Minard et al., 2013).

### Developmental Stage Affects Microbiota Composition

Our results suggest that the developmental stage influences microbiota composition in mosquitoes. Only one genus, *i.e.*, *Vulcaniibacterium*, was found to be shared by both Foshan and Crema samples. This bacterium has been previously found in the gut of both sexes of *Bactrocera oleae* (Koskinioti et al., 2019) but it was never detected in a mosquito species so far.

Microbacteriaceae, the only family found to be uniquely present in wild CR larval samples, was previously isolated from *Ae. albopictus* larvae and reported to be absent from the other developmental stages (Yadav et al., 2016). Similarly, in *Ae. aegypti*, Microbacteriaceae were found to be lost during metamorphosis (Frankel-Bricker et al., 2020). In our work, we confirmed the larval-specificity of this bacterial family in *Ae. albopictus*. While *Herbiconiux* was previously identified in *Ae. aegypti* larvae (Hery et al., 2021), and it is present also in our wild sampled larvae, *Leifsonia* was found in *Ae. albopictus* for the first time. This bacterium has been identified in the breeding site water of *A. darlingi* in Brazil, and in the midgut of the sandfly *Leishmania major* (Louradour et al., 2017), but to the extent of our knowledge it was never identified in *Ae. albopictus* before. The role of this bacterium in the microbiota of the tiger mosquito will require further investigation, extending the sampling to other natural breeding sites. *Leucobacer* was previously detected in the larvae of *Ae. aegypti* and were rare in breeding site water and nearly absent in adults (Coon et al., 2014), mirroring our results in *Ae. albopictus*.

### *Wolbachia* Abundance Contributes to Bacterial Community Structure

In addition to the effects of the breeding site environment, also interactions between members of the microbiota within a host can induce changes in the structure of the microbial communities and in the relative abundance of its components (Brinker et al., 2019). Recent studies began to show that the history of *Wolbachia* colonization has an impact on the physiological changes mediated by this bacterium in the host, with effects on the resident microbiota. After a stable transinfection, *Wolbachia* was shown to lead to a decrease in microbial diversity in *Ae. aegypti* (Audsley et al., 2018). This decrease in microbial diversity has been suggested to be mediated by immune system modulation, resource competition and the pH (Simhadri et al., 2017; Audsley et al., 2018). *Wolbachia*-mediated immune regulation appears to be lost in hosts developing long-term co-evolutionary relationships with this bacterium (Shi et al., 2018). Differently from what occurs in *Ae. aegypti*, *w*AlbA and *w*AlbB are native *Wolbachia* infections of *Ae. albopictus* with a long history of co-association (Dutton and Sinkins, 2004). Accordingly, here we show that *Ae. albopictus* adults displaying a high *Wolbachia* prevalence have a more diverse microbiota than mosquitoes with no or low *Wolbachia* reads. Our study did not focus on investigating whether and to what extent *Wolbachia* prevalence is linked to features of water at mosquito breeding sites. Further studies involving an extended sample size are needed to confirm results from our exploratory study as well as to further clarify the complex interactions between mosquito host, the environment and the different members of the bacterial communities residing in different tissues and organs, as mentioned above. The identification of *Wolbachia* also in somatic tissues of insects, including mosquitoes (see Pietri et al., 2016 for a review), led to formulate the hypothesis that this bacterium can also be acquired from the environment and/or host sharing, as suggested for ants and triatomines (Espino et al., 2009; Andersen et al., 2012; Frost et al., 2014).

### *Wolbachia* Prevalence in Laboratory Strains Varies

This study contributed to further support that *Wolbachia* is highly prevalent in *Ae. albopictus*. However, we found that in one strain from La Reunion Island (*i.e.*, Tampon), the prevalence was particularly low with respect to what detected in the other five strains and previously reported (Noor Afizah et al., 2015). Moreover, in the Crema strain derived from eggs collected in 2016 in the same site used for this study, we found that 3% of the mosquitoes were uninfected. Given that *Wolbachia* is transmitted vertically and this strain has been reared in our insectary for 33 generations, this result was unexpected and provides ground for further analyses.

Our results also confirmed the prevalence of dual *Wolbachia* infection in *Ae. albopictus* strains derived from different geographic populations. In the field, the prevalence of double infection by *Wolbachia* has been reported to be over 99.41% (Kittayapong et al., 2002a) and in Korean populations more than 98.8% (Park et al., 2016). We found dual infection ranging from 65 to 97%. As reported by previous studies, dual infection is mainly present in females (Joanne et al., 2015; Noor Afizah et al., 2015; Ahmad et al., 2017), but in two of the strains we analyzed, namely Crema and Tampon, both *Wolbachia* were present with higher frequency in males. A previous study that investigated *w*AlbA density in wild-sampled *Ae. albopictus* males from two Italian localities in the Central and Southern regions (*i.e.*, Central Italy: Crevalcore, Bologna; Southern Italy: Anguillara Sabazia, Rome) showed that *w*AlbA titer was very low in about half of the collected males from both sites (Calvitti et al., 2015), in agreement with the findings of Tortosa et al. (2010). However, density of both *Wolbachia* strains was confirmed to be impacted by adult age, population geographic origin and environmental conditions such as temperature and food availability in larval breeding sites (Calvitti et al., 2015).

Our data suggest that an extensive survey of *Wolbachia* prevalence in wild populations is extremely important not only to better understand the role of this bacterium in contributing to shape the microbial community in *Ae. albopictus*, but also to provide essential basic-biology information to inform current and future mosquito control programs based on the Incompatible Insect Technique approach, especially given that cytoplasmic incompatibility level has been correlated with *w*AlbA density (Calvitti et al., 2015).

## Data Availability Statement

The datasets generated for this study can be found in the European Nucleotide Archive (ENA) under the BioProject: PRJEB41031. The codes used for our analyses are available at the following link: https://gitlab.com/anna.sandionigi/metamosquito.

## Author Contributions

MB and MCas conceived and designed the experiment. FS, AS, MCar, and AB performed the experiments. FS, AS, and AB analyzed the data. FS, AS, AB, and MB wrote the manuscript. All the authors provided critical comments on the manuscript, read and approved the final version of the manuscript.

## Conflict of Interest

AS is employed by the company Quantia Consulting srl. The remaining authors declare that the research was conducted in the absence of any commercial or financial relationships that could be construed as a potential conflict of interest.

## Footnotes

1 http://bioinformatics.psb.ugent.be/webtools/Venn/

## Supplementary Material

Supplementary Figure 1 Water samples analyses. **(A)** 16S rDNA-based bacterial quantification in water samples. On the *X*-axis, water samples are reported, colored by sampling period (W Start – orange – and W End – green – refers to water collected at the moment of mosquito collection and after all adults had emerged, respectively). Values are expressed as log2(DNA counts)/mL. **(B)** Physical analysis of W Start and W End water samples. Shannon alpha diversity index, pH, and conductivity values are shown. Both pH and conductivity significantly increased in W End samples (*t*-test, *P* < 0.05).

Supplementary Figure 2 Rarefaction curves used to discover all the microbial ‘species’/ASVs in each sample where *Wolbachia* reads were removed.

Supplementary Figure 3 Bacterial genera shared between breeding site water, and wild and laboratory larvae and adults. Venn diagrams show the number of genera shared in **(A)** water (W Start vs. W End), **(B)** larval (CR vs. FO), and **(C)** adult (CR vs. FO) samples.

Supplementary Figure 4 Alpha diversity indices of adult individuals. Alpha diversity (Shannon) values distribution for adults (CR and FO) samples are shown. Each sample is indicated with a different color.

Supplementary Table 1 Number of sequencing reads per sample before and after removal of *Wolbachia*-associated reads. DNA extraction negative controls are included. Samples with no *Wolbachia* reads are indicated in bold.

Supplementary Table 2 List of ASVs identified in each larval, adult, and water sample.

Supplementary Table 3 Bacteria quantification through qPCR assays.

Supplementary Table 4 Physio-chemical analyses of water samples.

Supplementary Table 5 Proportion of sequencing reads assigned to a given bacterial taxon at the phylum and family level in water, larval, and adult samples. Only 50 most abundant ASVs were considered.

Supplementary Table 6 List of bacterial genera identified in the conserved microbiota for each larval, adult, and water sample.

Supplementary Table 7 List of unique and conserved bacterial genera from the microbiota of larvae and adults from wild or laboratory samples.
