# Supplementary figures and images for: Exploring Changes in the Microbiota of Aedes albopictus: Comparison Among Breeding Site Water, Larvae, and Adults
Source: Front Microbiol. 2021 Jan 28;12:624170. doi: 10.3389/fmicb.2021.624170 (PMC7876458; doi:10.3389/fmicb.2021.624170)

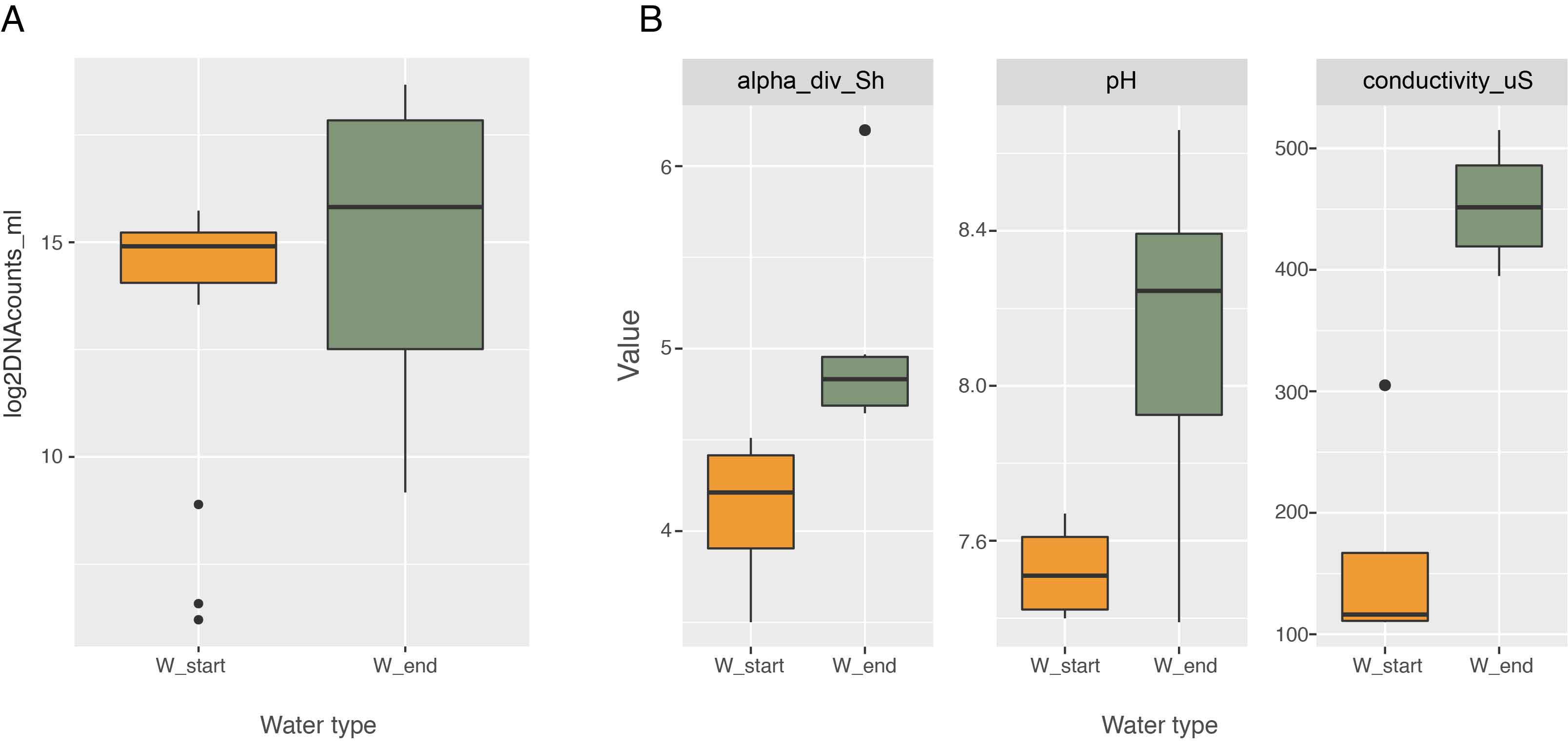

Supplement: Supplementary Figure 1 — Water samples analyses. (A) 16S rDNA-based bacterial quantification in water samples. On the X-axis, water samples are reported, colored by sampling period (W Start – orange – and W End – green – refers to water collected at the moment of mosquito collection and after all adults had emerged, respectively). Values are expressed as log2(DNA counts)/mL. (B) Physical analysis of W Start and W End water samples. Shannon alpha diversity index, pH, and conductivity values are shown. Both pH and conductivity significantly increased in W End samples (t-test, P < 0.05). [file Image_1.JPEG]

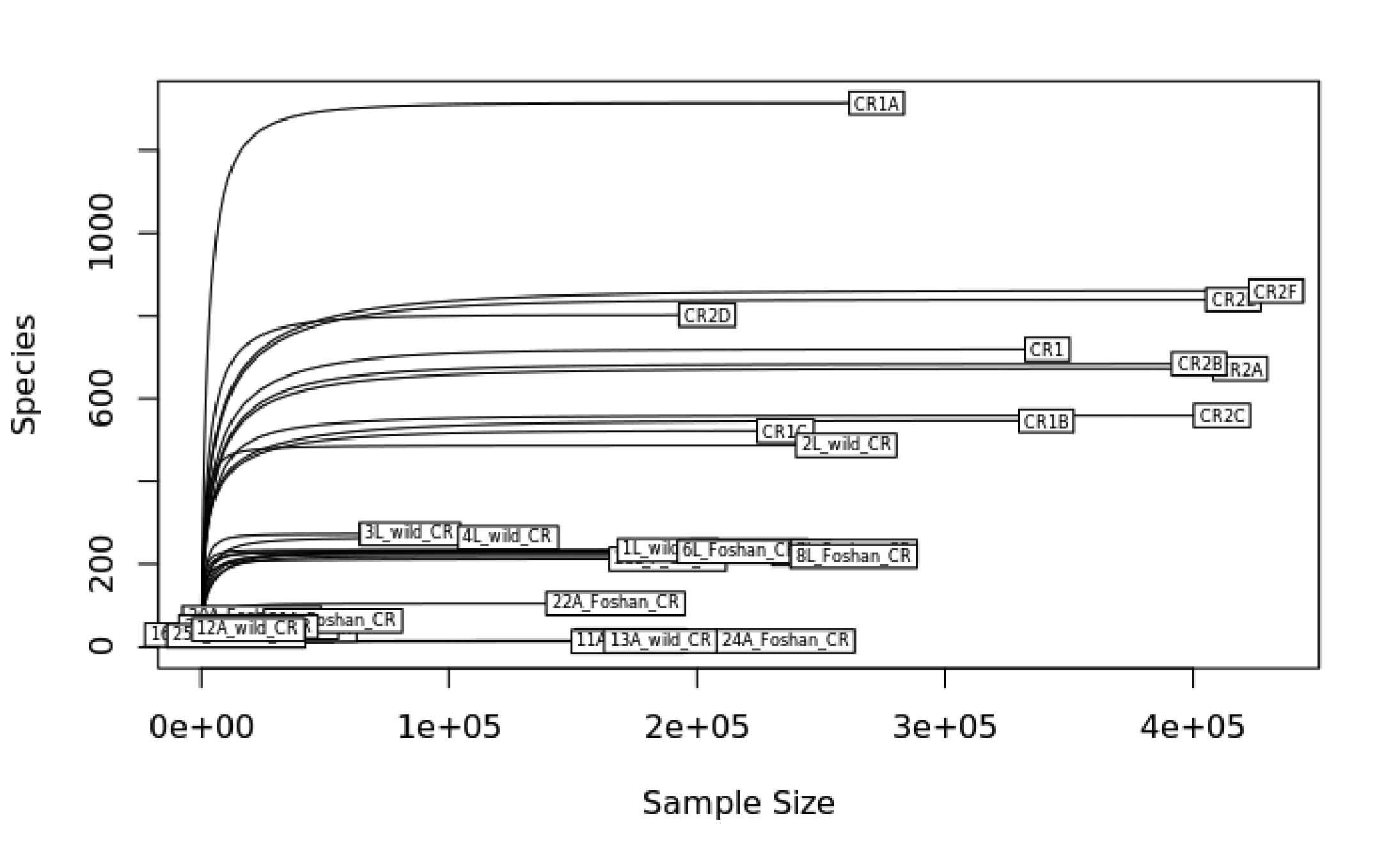

Supplement: Supplementary Figure 2 — Rarefaction curves used to discover all the microbial ‘species’/ASVs in each sample where Wolbachia reads were removed. [file Image_2.JPEG]

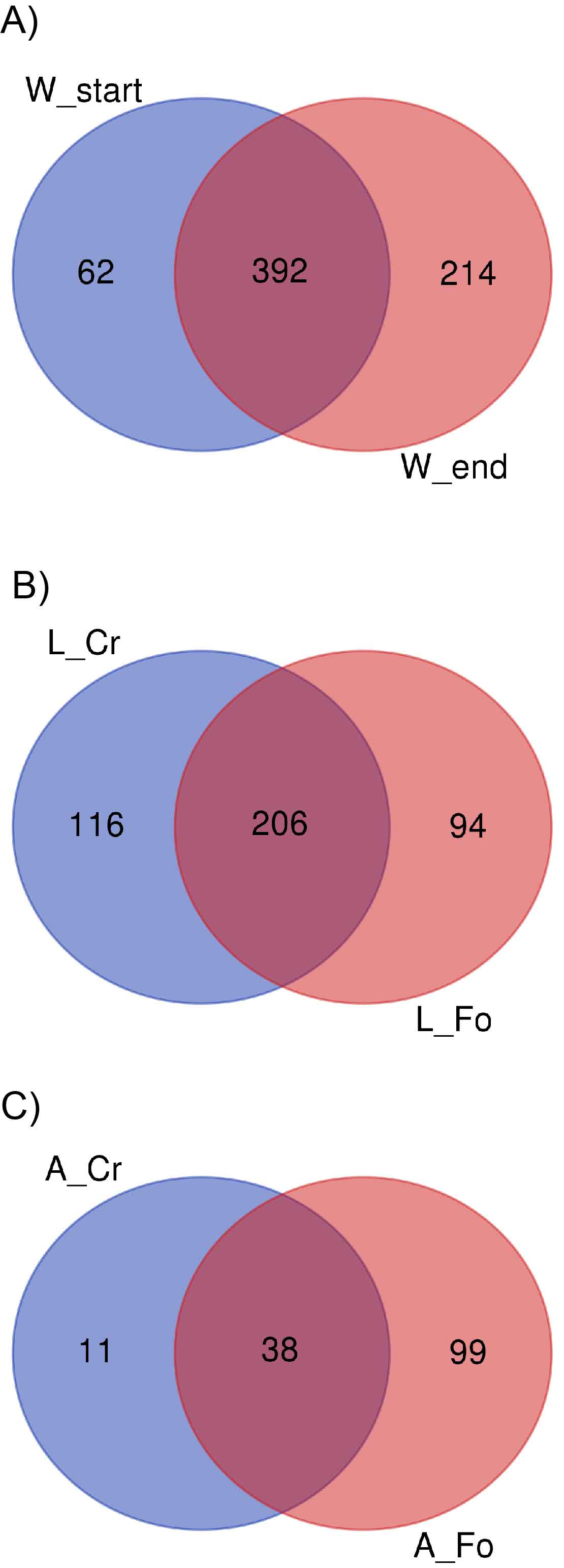

Supplement: Supplementary Figure 3 — Bacterial genera shared between breeding site water, and wild and laboratory larvae and adults. Venn diagrams show the number of genera shared in (A) water (W Start vs. W End), (B) larval (CR vs. FO), and (C) adult (CR vs. FO) samples. [file Image_3.JPEG]

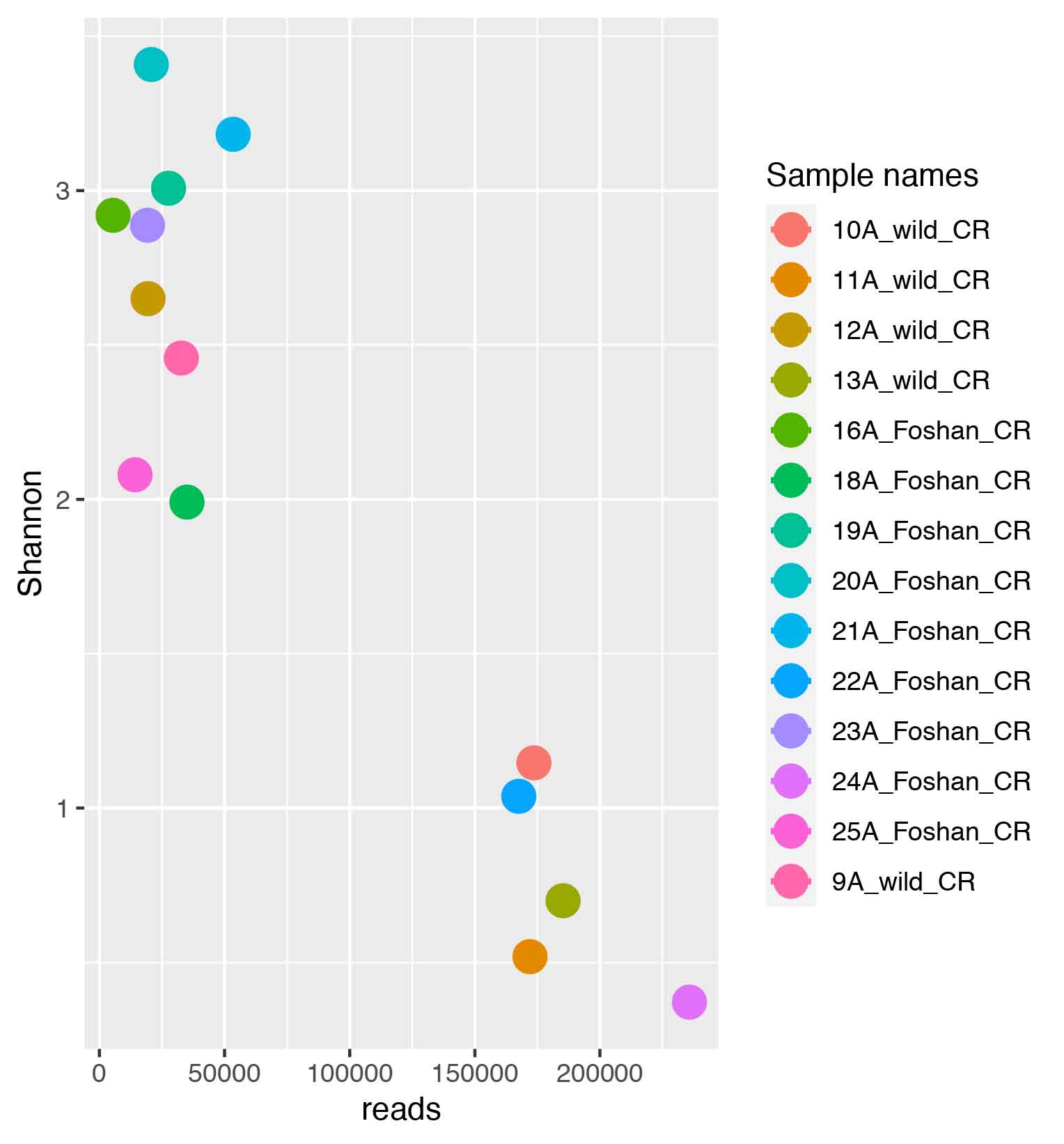

Supplement: Supplementary Figure 4 — Alpha diversity indices of adult individuals. Alpha diversity (Shannon) values distribution for adults (CR and FO) samples are shown. Each sample is indicated with a different color. [file Image_4.JPEG]
